# An amyloid beta vaccine that safely drives immunity to a key pathological species in Alzheimer’s disease: pyroglutamate amyloid beta

**DOI:** 10.1093/braincomms/fcac022

**Published:** 2022-02-04

**Authors:** M. Vukicevic, E. Fiorini, S. Siegert, R. Carpintero, M. Rincon-Restrepo, P. Lopez-Deber, N. Piot, M. Ayer, I. Rentero, C. Babolin, S. Bravo-Veyrat, V. Giriens, C. Morici, M. Beuzelin, A. Gesbert, S. Rivot, S. Depretti, P. Donati, J. Streffer, A. Pfeifer, M. H. Kosco-Vilbois

**Affiliations:** 1 AC Immune SA, Lausanne, Switzerland; 2 Biomedical Sciences, University of Antwerp, Antwerp, Belgium

**Keywords:** Alzheimer’s disease, vaccine, pyroglutamate amyloid beta, amyloid beta

## Abstract

Pyroglutamate amyloid beta3–42 (pGlu-Abeta3–42), a highly amyloidogenic and neurotoxic form of Abeta, is N-terminally truncated to form a pyroglutamate and has recently been proposed as a key target for immunotherapy. Optimized ACI-24, a vaccine in development for the treatment and prevention of Alzheimer’s disease, focuses the antibody response on the first 15 N-terminal amino acids of Abeta (Abeta1–15). Importantly, clinical data with an initial version of ACI-24 incorporating Abeta1–15, established the vaccine’s safety and tolerability with evidence of immunogenicity. To explore optimized ACI-24’s capacity to generate antibodies to pGlu-Abeta3–42, pre-clinical studies were carried out. Vaccinating mice and non-human primates demonstrated that optimized ACI-24 was well-tolerated and induced an antibody response against Abeta1–42 as expected, as well as high titres of IgG reactive with pyroGlu-Abeta. Epitope mapping of the polyclonal response confirmed these findings revealing broad coverage of epitopes particularly for Abeta peptides mimicking where cleavage occurs to form pGlu-Abeta3–42. These data are in striking contrast to results obtained with other clinically tested Abeta targeting vaccines which generated restricted and limited antibody diversity. Taken together, our findings demonstrate that optimized ACI-24 vaccination represents a breakthrough to provide a safe immune response with a broader Abeta sequence recognition compared to previously tested vaccines, creating binders to pathogenic forms of Abeta important in pathogenesis including pGlu-Abeta3–42.

## Teaser

Vukicevic *et al*. show that vaccination of mice or non-human primates with the optimized ACI-24 vaccine targeting amyloid beta1–15 (Abeta1–15) as epitope was well-tolerated and importantly induced immunoglobulin G antibodies recognizing not only Abeta1–15 but also the highly neurotoxic pyroglutamate Abeta species.

## Graphical Abstract

**Graphical Abstract:**
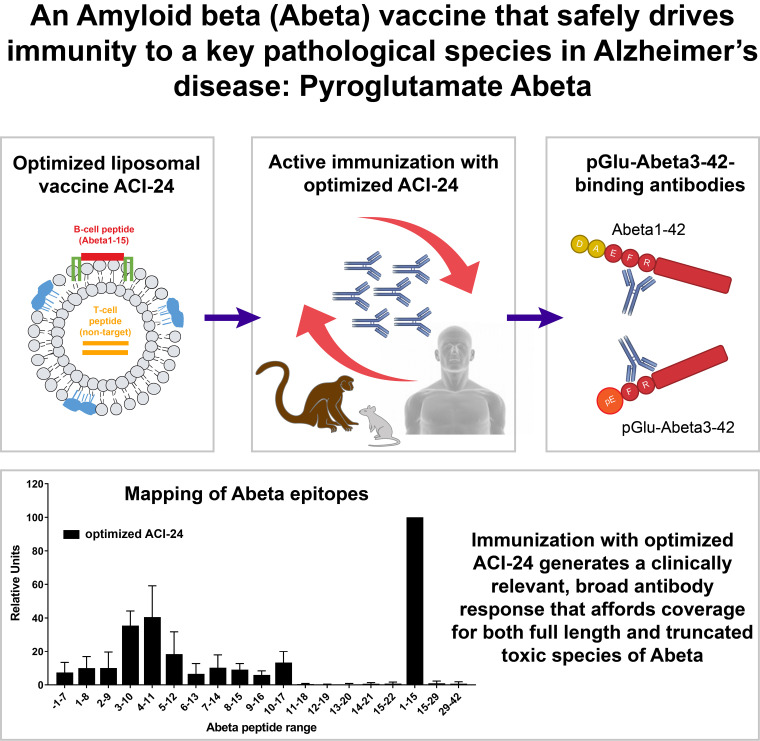

## Introduction

Alzheimer’s disease is a devastating, progressive neurodegenerative disorder characterized by the loss of cognitive function. Aamyloid beta (Abeta) deposition and the extracellular formation of senile plaques preceded by intraneuronal Abeta accumulation are associated with neuronal loss, vascular damage and Alzheimer’s dementia.^1,2^ In the natural, non-pathological form, Abeta is in a random-coil conformation. In the pathological state, the protein transforms into a secondary beta-sheet structure, spontaneously aggregating into insoluble deposits.^1^ While the most prominently studied species have been Abeta1–40 and Abeta1–42, shorter N-terminal cleavage forms have gained recent clinical attention such as pyroglutamate-modified Abeta (pGlu-Abeta3–42) due to an enhanced propensity to aggregate and provoke neurotoxicity.^3^ Indeed, of the various N-terminal modified Abeta peptides identified in Alzheimer’s disease brains, the most common is the pyroglutamate-modified.^4^ pGlu-Abeta3–42 accumulates in the brain before the appearance of clinical symptoms of Alzheimer’s disease, preceding Abeta1–42 deposition.^4^

pGlu-Abeta3–42 is formed by the cleavage of the first two N-terminal amino acids, aspartate and alanine, exposing glutamate at position 3 that is subsequently post-translationally modified to pyroglutamate. This form appears to be a vital constituent of senile plaques, highly amyloidogenic and neurotoxic.^5,6^ The N-terminal modification renders the variant highly stable, decreasing solubility and increasing aggregation susceptibility as compared to Abeta1–42 or Abeta1–40.^7,8^ Importantly, pGlu-Abeta3–42 was shown to act as a seed for template-induced misfolding^9^ which leads to its high amyloidogenicity. Consequently, this non-physiological pGlu-Abeta3–42 species is suggested to play a significant role in the neuronal toxicity of Alzheimer’s disease and represents an attractive target for therapy.^5^ Indeed, as a clinical proof of concept, passive immunization with the monoclonal antibody (mAb), Donanemab (LY3002813), which targets pGlu-Abeta3–42, demonstrated encouraging results in a recently published Phase 2 clinical study.^10^

Vaccination is a highly attractive paradigm for treatment and even prevention of Alzheimer’s disease. When cleverly formulated, a vaccine should safely induce an immune response consisting of a pool of diverse, target-specific, disease modifying antibodies. The initial step towards achieving this goal for Alzheimer’s disease involved the first in class vaccine, AN1792, in which the full-length sequence (1–42) of Abeta was used as the immunogen. AN1792 induced an antibody response that led to a promising clinical result with a slower rate of decline in patients who had received vaccination.^11^ However, 6% of these patients developed meningoencephalitis, an inflammatory reaction considered to be due to a T-cell-mediated response against full-length Abeta.^12^ Thus, a later vaccine, ACC-001, incorporated Abeta1–7 sequence to avoid inflammation as the first 15 amino acids of Abeta contains B-cell but no T-cell stimulating epitopes.^13^ Although in Phase 2a studies ACC-001 was observed to be well-tolerated and immunogenic, no effect was observed on cognitive decline.^14,15^

The initial version of ACI-24 and optimized ACI-24 are uniquely formulated vaccines that anchor the first 15 amino acids (1–15) of Abeta into a stacked beta-sheet conformation on the surface of liposomes. In pre-clinical studies conducted several years ago, the initial version of ACI-24 induced production of anti-Abeta antibodies specific for soluble and insoluble aggregated Abeta including oligomers with limited reactivity to monomers.^16–18^ These nonclinical results supported the evaluation of ACI-24 in three clinical studies where the vaccine has demonstrated favourable tolerability, immunogenicity and pharmacodynamic profiles (^19^ and manuscript submitted). Building on this knowledge of a safe and potentially highly effective Abeta1–15 targeting peptide as the B-cell epitope, an optimized formulation has been generated aiming to maximize the humoral response to the pathological target. For T-cell support, an additional set of peptides has been introduced into optimized ACI-24 that stimulate T-helper cells present in most adults. The peptide sequence originates from antigens to which humans are commonly exposed (e.g. tetanus). This approach has the advantage to obtain T-cell help without engaging Abeta-specific T-cells, ensuring an optimal anti-Abeta antibody response without the risk of developing an unwanted inflammatory reaction. Here, we present data from pre-clinical studies in mice and non-human primates that demonstrate the breadth of the polyclonal response generated by optimized ACI-24 vaccination for the truncated, pathological Abeta species, pGlu-Abeta3–42. In addition, we compare the ability of vaccines with other Abeta sequences as the immunogen to generate antibodies to pGlu-Abeta3–42, demonstrating a clear superiority of the immunogenic profile of optimized ACI-24.

## Materials and methods

### Animals

All animal studies were carried out in compliance with national and local directives on the protection of animals used for scientific purposes. For the studies involving mouse immunization, statistical analyses of previous experiments have shown that 10 mice per group are sufficient to observe statistically significant differences between groups. For studies, involving cynomolgus monkeys, the number of animals per group were chosen in compliance with the 3R principles (replace, reduce, refine).^20^

### Vaccines

Manufacturing details of all vaccines are provided in the Supplementary material. AN1792 and ACC-001 were generated as published.^14,21^

### Immunizations

Ten female C57BL/6J mice (Charles River, Italy) were immunized subcutanously (s.c.) with 200 µl of optimized ACI-24 corresponding to a dose of 80 µg of Abeta1–15 on Days 1 and 15. Blood samples were taken for plasma preparation 1 week prior to the first immunization and then 1 week after the second immunization (Day 22).

Four cynomolgus monkeys (two females and two males) were immunized intramuscularly (i.m.) with 2.5 ml of optimized ACI-24 (Abeta1–15 dose 1000 µg) on Days 0, 28, 56, 84 and 112. Blood samples were taken for serum preparation 1 week prior to the first immunization and then 1 week after the fifth immunization (Day 119).

Four cynomolgus monkeys (two females and two males) were immunized s.c. with 1 ml AN1792 (full-length Abeta1–42 dose 200 µg) mixed with 50 µg Quil-A® (InvivoGen, France) as adjuvant prior to the injection on Days 0, 28, 56 and 84. Blood samples were taken for serum preparation 1 week prior to the first immunization and then 1 week after the fourth immunization (Day 91).

Four cynomolgus monkeys (two females and two males) were immunized s.c. with 0.5 ml ACC-001 (CRM197-Abeta1–7 dose of 30 µg) mixed with QS-21 (50 µg, Desert King International, USA) prior to injection on Days 0, 28, 56, 84 and 112. Blood samples were taken for serum preparation 1 week prior to the first immunization and then 1 week after the fifth immunization (Day 119).

### Quantification of anti-Abeta1–42 or anti pyroglutamate Abeta3–42 antibodies by ELISA

The detailed procedure of the ELISA (enzyme-linked immunosorbent assay) is provided in the Supplementary material. Briefly, plates were coated overnight with either 10 µg/ml of Abeta1–42 or pGlu-Abeta3–42 peptide film. Mouse plasma or monkey sera were added and incubated at 37°C for 2 h. After washing, the detection antibody was incubated for 2 h at 37°C. The substrate was added and the optical density was read.

#### For mouse samples

The anti-Abeta1–42 or anti-pGlu-Abeta3–42 antibody concentrations were back-calculated against a calibration curve using the anti-Abeta mAb, 6E10 (BioLegend, UK). For the back calculation, a calibration fitting curve was determined using an unweighted four-parameter logistic (4PL) regression model using the Gen5 software (BioTek, Switzerland) with results expressed as ng/ml.

#### For monkey samples

The anti-Abeta1–42 or anti-pGlu-Abeta3–42 antibody concentrations were back-calculated against a calibration curve established using a pool of monkey serum created as a standard. As described for mouse samples, a 4PL calibration curve fitting was used but results expressed in arbitrary units (AU/ml).

### Epitope mapping

Epitope mapping to identify the binding site, or ‘epitope’, of the antibodies generated by the immunization was performed using CelluSpot peptide arrays (Intavis Peptide Services, Germany). Peptides of varying lengths were generated and attached to glass slides in duplicate according to the manufacturer’s instructions and published guidelines.^22^ The sequences of the Abeta peptides are provided in Supplementary Table 1, and the details of the procedure are described in the Supplementary Material section. Briefly, after overnight blocking, slides were incubated for 3 h with the mouse or monkey samples at RT. After washing, slides with monkey samples were incubated 1 h with a mouse anti-monkey immunoglobulin G (IgG) antibody (Thermofisher Scientific, Switzerland), followed by a donkey anti-mouse IgG coupled to IRDye800CW (LI-COR Biosciences, Switzerland). Slides with mouse samples were incubated with the donkey anti-mouse IgG-IRDye800CW antibody. Signals were visualized using the LI-COR Odyssey Infrared Imaging system using the 800 nm channel. Analysis was performed using the LI-COR Image Studio 5.0 software (grid array analysis function). The value for each sample was defined and reported as the average of the duplicate peptide array values. For data plotting, signals were scaled from 0 to 100 using a min–max normalization method (details provided in the Supplementary Material section).

### Statistical analysis

Data were analysed in GraphPad Prism 9. To compare IgG titre on Abeta1–42 or pGlu-Abeta3–42in mouse plasma, a Wilcoxon matched-pairs signed rank test was performed.

### Data availability statement

The authors confirm that the data supporting the findings of this study are available within the article, in its Supplementary Material, and/or from the corresponding author upon reasonable request.

## Results

**Figure 1:**
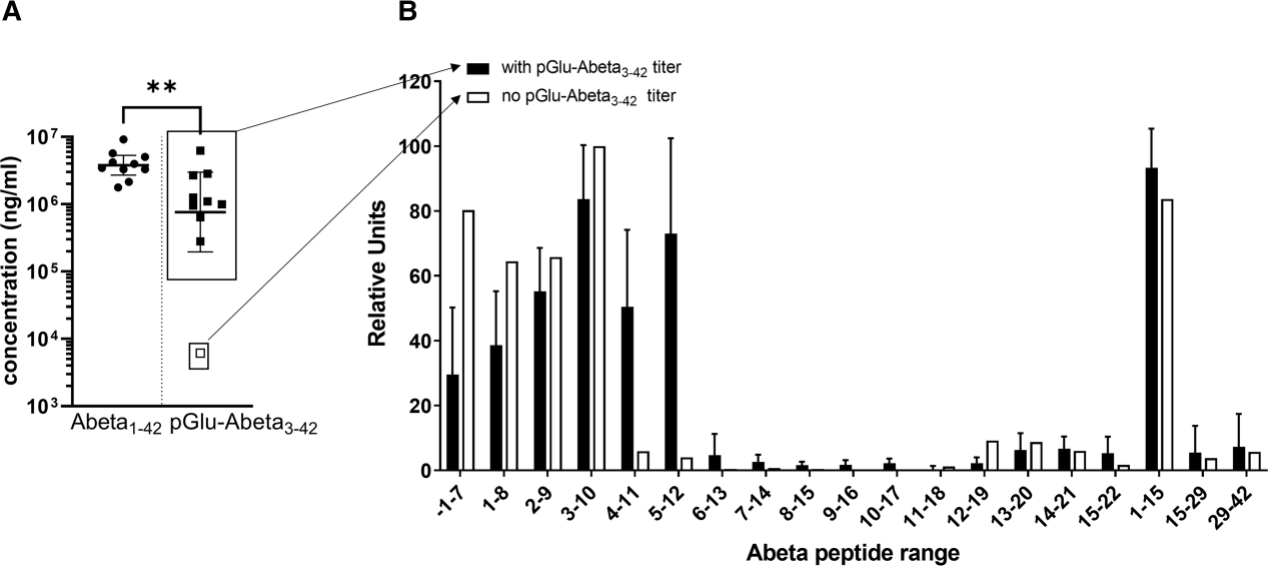
**Vaccination of mice with optimized ACI-24 induces a broad polyclonal response including epitopes present on full-length Abeta1–42 and the truncated pGlu-Abeta3–42 species**. C57BL/6J mice (*n* = 10) were immunized s.c. with optimized ACI-24 on Days 1 and 15 and plasma samples collected on day 22. (**A**) Determination of IgG titres binding to Abeta1–42 or pGlu-Abeta3–42 was carried out by an ELISA. IgG levels from samples obtained prior to vaccination were below the LLOQ (6130 ng/ml). Individual points represent the back-calculated IgG concentration in ng/ml per animal, lines show the geometric mean  ± 95% confidence interval per group. Wilcoxon matched-pairs signed rank test was performed to assess statistical significance. **P < 0.01 Dashed line indicates LLOQ of the assay; (**B**) Mapping of the Abeta epitopes of the polyclonal response for each mouse was performed using arrays containing peptides with a shift of one amino acid of Abeta1–22. A peptide containing the first amino acid flanking the N-terminus of the Abeta region within the APP protein sequence was also included in the array (peptide −1–7). The peptide used as the immunogen, i.e. Abeta1–15, was used as a positive control. To assess binding outside of the target vaccine antigen, Abeta 15–29 and Abeta29–42 were used. The means ± standard deviations (SDs) of the rescaled signals (relative units) are shown. Solid bars, the nine mice with strong binding, and hatched bars, the single mouse with very low binding to pGlu-Abeta3–42. To normalize the values, mathematical rescaling was done based on the maximal and minimal obtained value per mouse multiplied by 100.

Mice were vaccinated s.c. on Days 1 and 15, and antigen-specific plasma titres measured prior to and at 7 days after the second immunization with optimized ACI-24. All mice (i.e. 10/10) produced a highly consistent, anti-Abeta1–42 IgG response several logs higher than baseline already 7 days after the second vaccination (Fig. 1A). For the IgG levels capable of binding to the truncated fragment, pGlu-Abeta3–42, nine of 10 mice produced antibody titres (Fig. 1A) 2–4 logs higher than pre-vaccination levels which were below the lower limit of quantification (LLOQ) for both assays and for all mice (data not shown).

Next, we characterized the antibody binding sites by epitope mapping. As shown in Fig. 1B, the profile of the plasma with high binding titres for pGlu-Abeta3–42 demonstrated a broad coverage of Abeta epitopes including a substantial proportion of IgGs that recognized the Abeta4–11 and Abeta5–12 peptides. In contrast, for the one mouse with very low binding signal to pGlu-Abeta3–42, the profile was focused on the N-terminal sequence as the presence of amino acid 3 in the Abeta peptide was crucial for recognition. These data indicate that optimized ACI-24 induces a favourable broad panel of antibodies that recognizes not just the N-terminal Abeta peptide sequences but also species that would be truncated such as the neurotoxic pGlu-Abeta3–42.

**Figure 2:**
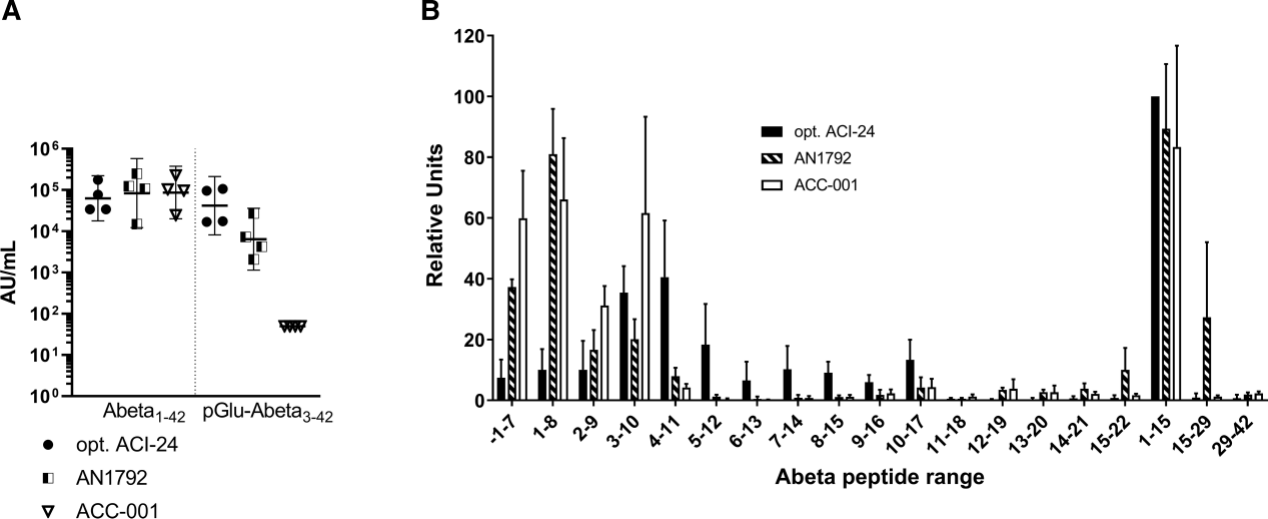
**Vaccination of non-human primates with optimized ACI-24 induces a potent and broad polyclonal response to epitopes present on full-length Abeta1–42 and the truncated pGlu-Abeta3–42 species**. Cynomolgus monkeys were immunized with the Abeta vaccines, optimized ACI-24, AN1792 and ACC-001, and serum collected for determination of anti-Abeta and anti-pGlu-Abeta3–42 IgG titres and the binding preference of the immune polyclonal sera. (**A**) Determination of IgG titres binding to Abeta1–42 or pGlu-Abeta3–42 was carried out by an ELISA. Individual points show the back-calculated IgG concentration in AU/ml per animal, also the geometric mean and the 95% confidence interval is shown for each group. Due to the standard limited sample size when conducting studies with non human primates no meaningful analysis could be performed to assess statistical differences between the different groups. (**B**) Mapping of the Abeta epitopes of the polyclonal response for each monkey was performed using arrays containing peptides with an offset of one amino acid of Abeta1–22. A peptide containing the first amino acid flanking the N-terminus of the Abeta region within the APP protein sequence was also included in the array (peptide −1–7). The peptide used as the immunogen, i.e. Abeta1–15, was used as a positive control. To assess binding outside of the target vaccine antigen, Abeta 15–29 and Abeta29–42 were assessed. The means ± standard deviations (SDs) of the rescaled signals (relative units) are shown. To normalize the values, mathematical rescaling was done based on the maximal and minimal obtained value per monkey multiplied by 100.

Following these encouraging results in mice, we assessed the immunogenicity of optimized ACI-24 in non-human primates. Four cynomolgus monkeys were i.m. on Days 0, 28, 56, 84 and 112. Serum samples were obtained prior to vaccination and 7 days post the fifth injection, i.e. Day 119. All monkeys vaccinated with optimized ACI-24 produced a strong IgG response to Abeta1–42 (33 909–176 695 AU/ml) and pGlu-Abeta3–42 (16 951–106 662 AU/ml) (Fig. 2A). Importantly, the vaccine was very well-tolerated with no adverse events reported beyond transient, local injection site reactions that spontaneously resolved.

AN1792 and ACC-001, two vaccines previously tested in clinical trials, were also used to vaccinate cynomolgus monkeys. Serum samples were collected for AN1792 prior to vaccination and on Day 91 after s.c. injection on Days 0, 28, 56 and 84. For ACC-001, serum samples were collected prior to vaccination and on Day 119 after s.c. injection on Days 0, 28, 56, 84 and 112. Interestingly, both vaccines generated comparable IgG titres to Abeta1–42 (Fig. 2A; AN1792: 14 843–250 039 AU/ml, ACC-001 24 629–226 920 AU/ml). However, AN1792 produced only a modest level of antibodies (2071–27 004 AU/ml) able to bind to pGlu-Abeta3–42 as compared to optimized ACI-24 despite using the full-length Abeta1–42 as the immunogen. Even more strikingly, the sera from monkeys immunized with ACC-001 demonstrated a poor capacity to bind to pGlu-Abeta3–42, which could be directly related to the presentation of the immunogen, i.e. Abeta1–7.

Epitope mapping was then carried out on all serum samples to better understand and compare the coverage and specificity of the polyclonal response generated by the three vaccines (Fig. 2B). Similar to the results when vaccinating mice, optimized ACI-24 induced IgGs with a broad recognition pattern of the Abeta peptide sequences. Binding was observed for all epitopes between the N-terminal peptides through Abeta8–17 with the largest signal for Abeta3–10, Abeta4–11 and Abeta5–12. In contrast, the vaccines, AN1792 and ACC-001, induced IgGs predominantly recognizing the N-terminal peptides, with very little or no recognition of Abeta4–11 and Abeta5–12 peptides.

## Discussion

Here, we describe the results of pre-clinical studies conducted to examine the antibody response to pathological Abeta of the vaccine, optimized ACI-24. When used to immunize mice and non-human primates, optimized ACI-24 demonstrates a strong antibody response to Abeta1–42 as expected, as well as pGlu-Abeta3–42, forms of Abeta that have been demonstrated to be highly amyloidogenic and neurotoxic, driving Alzheimer’s disease progression.

Antibody epitope mapping confirmed and provided an explanation for these observations of the polyclonal antibody response. The generation of substantial IgG titres that can be maintained over time (i.e. over 4 months) to pathological and clinically relevant forms of Abeta in non-human primates as well as mice attest to the potentially unique formulation of this vaccine that presents the B-cell epitope as an anchored protein forming a beta-sheet structure. These data build on the results originally published by Muhs *et al.*^16^ demonstrating that the initial vaccine version, also using Abeta1–15 as the target immunogen, preferentially generated antibodies against amyloid sequences in a beta-sheet conformation with increased affinity for aggregated beta-amyloid.^16,18^

Furthermore, this suggestion of potential superiority among Abeta targeting vaccines is supported by the data presented here for AN1792 and ACC-001 demonstrating limited Abeta species coverage. Indeed, our data align with published results for epitope mapping of post-vaccination, human clinical samples in which the tested patient samples displayed a dominant propensity for recognition of Abeta1–10, whereas samples from only two patients recognized Abeta2–11 and no samples were capable of binding to Abeta3–12.^21^ For ACC-001, data available from our study demonstrate that the induced antibodies are capable of recognizing the full-length Abeta1–42, however, not the truncated form, pGlu-Abeta3–42.

Taken together, these data illustrate that optimized ACI-24 vaccination generates IgGs with a broader Abeta sequence recognition compared with previously used vaccines. The binding to pathogenic forms of Abeta including truncated pGlu-Abeta3–42 species have been speculated to be clinically important in Alzheimer’s disease.^5^ This notion is now receiving clinical validation as a proof of concept study involving passive immunization with a mAb, Donanemab (LY3002813), specific for pyroglutamate Abeta, has demonstrated encouraging clinical results in a recently published Phase 2 clinical study.^10^

The core structure of optimized ACI-24 is based on nanosized lipidic bilayer structures (liposomes) as an antigen-carrying vehicle. The advantages of liposomes as a vaccine delivery system are their ability to protect antigens against degradation and carry single or multiple hydrophilic and lipophilic antigens. In addition, when anchored with palmitoyl chains, as is the case for optimized ACI-24, the Abeta1–15 antigenic peptide is presented on the surface of the liposomes as beta-sheet structures, which are in the conformational format that mimics the pathological form of the protein.^16–18^ The effect of presenting the conformational structure to the immune system is to significantly enhance the crosslinking and activation of B-cell receptors specific for the pathological form of Abeta, promoting increased cellular uptake of the immunogen and ultimately, improved antigen-specific antibody responses to the pathological forms of Abeta.^16–18^

The original formulation of ACI-24 has completed Phase 1 and Phase 2 studies in patients with mild Alzheimer’s disease, as well as a Phase 1b randomized, placebo controlled dose escalation study of the safety, tolerability and immunogenicity in Adults with Down syndrome (manuscript submitted). In all three clinical trials, ACI-24 was considered safe and well-tolerated with evidence of immunogenicity. In addition, no observations of toxicity were observed in the studies conducted here, outside of the transient local injection reactions (manuscript in preparation). Thus, importantly, anti-Abeta antibody titres have not been associated with any adverse findings in relevant animal species or humans. These results support the progression to clinical trials with the optimized formulation of ACI-24.

Taken together, a vaccine, such as optimized ACI-24 represents a powerful alternative to passive immunotherapy. The endogenous polyclonal antibody response of the subject provides a plethora of antibodies against various epitopes that can engage more than just one species, that will lead to better clearance of pathological proteins. In addition, the schedule of administration can be ultimately substantially less frequent once initial vaccination is completed and protective immunity established. In conclusion our data provide evidence, that the optimized ACI-24 can elicit a safe and potent immune response, with a preference towards pathologic forms of Abeta, providing an alternative to mAb-based therapies which are expensive and difficult to provide as a prevention strategy on a global scale.

## Supplementary Material

fcac022_Supplementary_Data

## Abbreviations

Abeta =: amyloid beta
IgG =: immunoglobulin G
i.m. =: intramuscularly
LLOQ =: lower limit of quantification
mAb =: monoclonal antibody
pGlu-Abeta3–42 =: pyroglutamate amyloid beta3–42
s.c. =: subcutanously

## References

[1] Hardy JA, Higgins GA. Alzheimer’s disease: The amyloid cascade hypothesis. Science. 1992;256(5054):184–185. 1566067 10.1126/science.1566067

[2] Wirths O, Multhaup G, Bayer TA. A modified beta-amyloid hypothesis: Intraneuronal accumulation of the beta-amyloid peptide–the first step of a fatal cascade. J Neurochem. 2004;91(3):513–520. 15485483 10.1111/j.1471-4159.2004.02737.x

[3] Pike CJ, Overman MJ, Cotman CW. Amino-terminal deletions enhance aggregation of beta-amyloid peptides in vitro. J Biol Chem. 1995;270(41):23895–23898. 7592576 10.1074/jbc.270.41.23895

[4] Sofola-Adesakin O, Khericha M, Snoeren I, Tsuda L, Partridge L. pGluAbeta increases accumulation of Abeta in vivo and exacerbates its toxicity. Acta Neuropathol Commun. 2016;4(1):109. 27717375 10.1186/s40478-016-0380-x PMC5055666

[5] Jawhar S, Wirths O, Bayer TA. Pyroglutamate amyloid-beta (Abeta): A hatchet man in Alzheimer disease. J Biol Chem. 2011;286(45):38825–38832. 21965666 10.1074/jbc.R111.288308 PMC3234707

[6] Cynis H, Frost JL, Crehan H, Lemere CA. Immunotherapy targeting pyroglutamate-3 Abeta: Prospects and challenges. Mol Neurodegener. 2016;11(1):48. 27363697 10.1186/s13024-016-0115-2 PMC4929720

[7] Schlenzig D, Manhart S, Cinar Y, et al Pyroglutamate formation influences solubility and amyloidogenicity of amyloid peptides. Biochemistry. 2009;48(29):7072–7078. 19518051 10.1021/bi900818a

[8] Schlenzig D, Ronicke R, Cynis H, et al N-terminal pyroglutamate formation of Abeta38 and Abeta40 enforces oligomer formation and potency to disrupt hippocampal long-term potentiation. J Neurochem. 2012;121(5):774–784. 22375951 10.1111/j.1471-4159.2012.07707.x

[9] Nussbaum JM, Schilling S, Cynis H, et al Prion-like behaviour and tau-dependent cytotoxicity of pyroglutamylated amyloid-beta. Nature. 2012;485(7400):651–655. 22660329 10.1038/nature11060 PMC3367389

[10] Mintun MA, Lo AC, Duggan Evans C, et al Donanemab in early Alzheimer’s disease. N Engl J Med. 2021;384:1691–1704. 33720637 10.1056/NEJMoa2100708

[11] Gilman S, Koller M, Black RS, et al Clinical effects of Abeta immunization (AN1792) in patients with AD in an interrupted trial. Neurology. 2005;64(9):1553–1562. 15883316 10.1212/01.WNL.0000159740.16984.3C

[12] Orgogozo JM, Gilman S, Dartigues JF, et al Subacute meningoencephalitis in a subset of patients with AD after Abeta42 immunization. Neurology. 2003;61(1):46–54. 12847155 10.1212/01.wnl.0000073623.84147.a8

[13] Monsonego A, Weiner HL. Immunotherapeutic approaches to Alzheimer’s disease. Science. 2003;302(5646):834–838. 14593170 10.1126/science.1088469

[14] Pasquier F, Sadowsky C, Holstein A, et al Two phase 2 multiple ascending-dose studies of vanutide cridificar (ACC-001) and QS-21 adjuvant in mild-to-moderate Alzheimer’s disease. J Alzheimers Dis. 2016;51(4):1131–1143. 26967206 10.3233/JAD-150376

[15] Hull M, Sadowsky C, Arai H, et al Long-term extensions of randomized vaccination trials of ACC-001 and QS-21 in mild to moderate Alzheimer’s disease. Curr Alzheimer Res. 2017;14(7):696–708. 28124589 10.2174/1567205014666170117101537 PMC5543567

[16] Muhs A, Hickman DT, Pihlgren M, et al Liposomal vaccines with conformation-specific amyloid peptide antigens define immune response and efficacy in APP transgenic mice. Proc Natl Acad Sci USA. 2007;104(23):9810–9815. 17517595 10.1073/pnas.0703137104 PMC1887581

[17] Pihlgren M, Silva AB, Madani R, et al TLR4- and TRIF-dependent stimulation of B lymphocytes by peptide liposomes enables T cell-independent isotype switch in mice. Blood. 2013;121(1):85–94. 23144170 10.1182/blood-2012-02-413831

[18] Hickman DT, Lopez-Deber MP, Ndao DM, et al Sequence-independent control of peptide conformation in liposomal vaccines for targeting protein misfolding diseases. J Biol Chem. 2011;286(16):13966–13976. 21343310 10.1074/jbc.M110.186338 PMC3077597

[19] Rafii MS. Alzheimer’s disease in down syndrome: Progress in the design and conduct of drug prevention trials. CNS Drugs. 2020;34(8):785–794. 32506291 10.1007/s40263-020-00740-6 PMC7395870

[20] Prior H, Sewell F, Stewart J. Overview of 3Rs opportunities in drug discovery and development using non-human primates. Drug Discov Today Dis Models. 2017;23:11–16.

[21] Lee M, Bard F, Johnson-Wood K, et al Abeta42 immunization in Alzheimer’s disease generates Abeta N-terminal antibodies. Ann Neurol. 2005;58(3):430–435. 16130106 10.1002/ana.20592

[22] Carter JM, Loomis-Price L. B cell epitope mapping using synthetic peptides. Curr Protoc Immunol. 2004;Chapter 9:Unit 9 4. 10.1002/0471142735.im0904s60 18432936

