# Supplementary material for: An amyloid beta vaccine that safely drives immunity to a key pathological species in Alzheimer’s disease: pyroglutamate amyloid beta
Source: Brain Commun. 2022 Feb 4;4(1):fcac022. doi: 10.1093/braincomms/fcac022 (PMC9037369; doi:10.1093/braincomms/fcac022)
Supplement: fcac022_Supplementary_Data [file fcac022_Supplementary_Data.docx]

**Supplementary Materials**

### Supplementary Materials and Methods

### Vaccines

Optimized ACI-24 was generated by dissolving lipids DMPC (= 1,2-dimyristoyl-sn-glycero-3-phospho-choline), DMPG (= 1,2-dimyristol-sn-glycero-3-[phosphor-rac(1-glycerol)] sodium salt), cholesterol and 3D(6-acyl) PHAD®, Monophosphoryl Hexa-acyl Lipid A, 3-Deacyl (Synthetic) (Avanti Polar Lipids, USA)in ethanol and the T cell peptide solubilized in histidine and sucrose before mixing using a crossflow injection module to form intermediate liposomes. The liposomes were then subjected to repeated extrusion cycles with a final ultra-/diafiltration step, filtered through a 0.2 µm filter and stored at 4°C until use.

AN1792 was generated according to reference^21^. Briefly, Abeta1-42 peptide (Bachem, Switzerland) was solubilized in water (2.2mg/mL) by vortexing for 3min. 10X PBS (Sigma, Switzerland) was added to maintain a concentration of 1X PBS in the final formulation. Complete dissolution of the Abeta1-42 peptide was achieved by 18h incubation at 37°C. Tween-80 was added (final concentration 0.8%) and the final Abeta1-42 (460µg/ml) stored at 2-8°C until use and mixed with 50µg Quil-A® (InvivoGen, France) prior to injection.

ACC-001 was generated according to reference^14^. Briefly, the carrier protein, Cross Reactive Material 197 (CRM197), was conjugated to the C-terminus of Abeta1-7 using H-DAEFRHD-Cys(CRM197-SBAP)-NH2 (SBAP = succinimidyl-3(bromoacetamido)propionate (Pepscan, Netherlands). ACC-001 was stored at -20°C until use and mixed with QS-21 (50µg; Desert King International, USA) prior to injection.

### Peptide film preparation of Abeta1-42 or pGlu-Abeta3-42 for ELISA

A peptide film to be used as the coating material for use in an ELISA was prepared by resuspending Abeta1-42 (Bachem, Switzerland) or pGlu-Abeta3-42 (Bachem, Switzerland) in 1,1,1,3,3,3-hexafluoro-2-propanol (HFIP; Sigma, Switzerland) to a final concentration of 1mM. After sonication for 15min, the solution was agitated overnight on a plate shaker at room temperature. Single-use aliquots were prepared and the supernatant evaporated for 2h under vacuum, then stored at -80°C until use.

### Quantification of anti-Abeta1-42 or anti-pGlu Abeta3-42 antibodies by ELISA

Plates were coated overnight at 4°C with either 10µg/mL of Abeta1-42 or pGlu-Abeta3-42 peptide film (described above). After washing with 0.05% Tween in PBS (Sigma, Switzerland) and blocking with 1% BSA (Sigma, Switzerland) in 0.05% Tween PBS, eight two-fold serial dilutions of mouse plasma or monkey sera starting at a 1:100 dilution were added and incubated at 37°C for two hours. After washing, the detection antibody (for mouse: alkaline phosphatase coupled anti-mouse IgG; for monkey: peroxidase conjugated anti-human/monkey IgG; Jackson ImmunoResearch, UK) was incubated for two hours at 37°C. After a final wash, plates were incubated with the substrate as described below:

For mouse samples: plates were incubated for 2.5 hours with the substrate pNPP dissolved in 5mL substrate diluent solution (0.1M Glycine, 1mM magnesium chloride and 1mM zinc chloride adjusted to pH 10.4; all from Sigma, Switzerland) and afterwards read at 405nm in an ELISA plate reader (SYNERGY HTX, BioTek/Agilent, Switzerland). The anti-Abeta1-42 or anti-pGlu-Abeta3-42 antibody concentrations were back-calculated against a calibration curve established using eight two-fold serial dilutions of the anti-Abeta monoclonal antibody (mAb), 6E10 (BioLegend, UK) in a starting concentration of 10µg/mL. As a quality control the anti-Abeta17-24 mAb, 4G8 (BioLegend, UK), was analyzed in eight two-fold dilutions.

For the back calculation, a calibration curve was determined using an unweighted four-parameter logistic (4PL) regression model using the Gen5 software (BioTek, Switzerland) with results expressed as ng/mL. The concentration was calculated for each sample dilution with O.D. (optical density) values between 0.8 and 2.8, the mean of all different dilutions was reported.

For monkey samples: plates were incubated for one hour with ABTS in H2O2, Roche Diagnostics (Sigma, Switzerland); H2O2 (MERCK, Switzerland) and afterwards read at 405nm in an ELISA plate reader (SYNERGY HTX, BioTek/Agilent, Switzerland). The obtained anti-Abeta1-42 or anti-pGlu-Abeta3-42 antibody concentrations were back-calculated against a calibration curve established using eight two-fold serial dilutions of a pool of monkey serum created as a standard. The pool was produced by combining the serum samples from 2 cynomolgus monkeys obtained at different times post-immunization with ACI-24.

As described for mouse samples, a 4PL calibration curve fitting was used, and results expressed in arbitrary units (AU/mL). The concentration was calculated for each sample dilution for O.D. between 0.3 and 3.0, and the mean of the different dilutions reported.

### Antibody epitope mapping

Antibody epitope mapping was carried out using synthetic peptides in conjunction with CelluSpot peptide arrays (Intavis Peptide Services, Germany)^22^. For this, various peptides were generated (Supplementary Table 1). To assess the binding to regions associated with the target antigen, arrays were generated with peptides 1-17 covering amino acids of Abeta 1-22 (Supplementary Table 1) including a sequence with -1-7 (i.e., peptide 1) representing the N-terminal flanking amino acid within the Amyloid beta precursor protein (APP). In addition, to assess antibody binding outside of the vaccine’s target antigen, Abeta15-29 and Abeta29-42 were used. The arrays were prepared in duplicate on glass slides. Slides were incubated overnight with Intercept blocking solution (LI-COR, Switzerland; 2-fold diluted in PBS), followed by a 3h incubation at RT with the mouse or monkey samples diluted in blocking buffer (0.2% Tween (Sigma, Switzerland) in Intercept blocking solution) in a humidified chamber. After washing in 0.05%Tween/PBS buffer, the slides with monkey samples were incubated for 1 hour with a mouse anti-monkey IgG antibody (Thermofisher Scientific, Switzerland), followed by a 1h incubation with a donkey anti-mouse IgG coupled to IRDye800CW (LI-COR Biosciences, Switzerland), both antibodies diluted in blocking buffer, while slides with mouse samples were only incubated with the donkey anti-mouse IgG-IRDye800CW detection antibody. Incubations were performed at RT under agitation on an orbital shaker (Heidolph, Germany).

After a final washing step, signals were visualized using the LI-COR Odyssey Infrared Imaging system using the 800nm channel. Analysis was performed using the LI-COR Image Studio 5.0 software (grid array analysis function). The value for each sample was defined as the average of the duplicate peptide array signals.

For reporting, all signals were defined as relative units by scaling the measured signal from 0 to 100 using a min-max normalization method:

Minimum (y(min)) and maximum values (y(max)) were calculated from each peptide array, and a new value was assigned using the following formula:

$$y^{'}=\frac{y-y\left( \min\right)}{y\left( \max\right)-y\left( \min\right)}x100$$

Where y’ (in relative units) is the normalized, rescaled signal, and y the measured signal.

**Supplementary Table 1: Abeta peptide sequences used for generating the epitope mapping arrays**

| **Peptide**  **number** | **Designated nomenclature**  **of Abeta peptides*** | **Sequence** |
| --- | --- | --- |
| **1** | -1-7 | MDAEFRHD |
| **2** | 1-8 | DAEFRHDS |
| **3** | 2-9 | AEFRHDSG |
| **4** | 3-10 | EFRHDSGY |
| **5** | 4-11 | FRHDSGYE |
| **6** | 5-12 | RHDSGYEV |
| **7** | 6-13 | HDSGYEVH |
| **8** | 7-14 | DSGYEVHH |
| **9** | 8-15 | SGYEVHHQ |
| **10** | 9-16 | GYEVHHQK |
| **11** | 10-17 | YEVHHQKL |
| **12** | 11-18 | EVHHQKLV |
| **13** | 12-19 | VHHQKLVF |
| **14** | 13-20 | HHQKLVFF |
| **15** | 14-21 | HQKLVFFA |
| **16** | 15-22 | QKLVFFAE |
| **17** | 1-15 | DAEFRHDSGYEVHHQ |
| **18** | 15-29 | QKLVFFAEDVGSNKG |
| **19** | 29-42 | GAIIGLMVGGVVIA |

The nomenclature refers to the amino acid number in the Abeta protein sequence with -1 representing the N-terminal flanking amino acid within the precursor protein APP
